# Neglected human *Rickettsia felis* infection in Taiwan: A retrospective seroepidemiological survey of patients with suspected rickettsioses

**DOI:** 10.1371/journal.pntd.0009355

**Published:** 2021-04-19

**Authors:** Wan-Hsiu Yang, Meng-Shiuan Hsu, Pei-Yun Shu, Kun-Hsien Tsai, Chi-Tai Fang

**Affiliations:** 1 Institute of Epidemiology and Preventive Medicine, College of Public Health, National Taiwan University, Taipei, Taiwan; 2 Department of Internal Medicine, Section of Infectious Disease, Far Eastern Memorial Hospital, Taipei, Taiwan; 3 Department of Healthcare Administration, Oriental Institute of Technology, Taipei, Taiwan; 4 Center for Diagnostics and Vaccine Development, Centers for Disease Control, Ministry of Health and Welfare, Taipei, Taiwan; 5 Institute of Environmental and Occupational Health Sciences, College of Public Health, National Taiwan University, Taipei, Taiwan; 6 Department of Public Health, College of Public Health, National Taiwan University, Taipei, Taiwan; 7 Division of Infectious Diseases, Department of Internal Medicine, National Taiwan University Hospital, Taipei, Taiwan; Baylor College of Medicine, UNITED STATES

## Abstract

**Background:**

Current knowledge on *Rickettsia felis* infection in humans is based on sporadic case reports. Here we conducted a retrospective seroepidemiological survey of *R*. *felis* infection among febrile patients visiting a medical center in Taipei.

**Methodology/Principal findings:**

A total of 122 patients with suspected rickettsioses presenting with fever of unknown origin (FUO) but tested negative for scrub typhus, murine typhus, or Q fever were retrospectively identified during 2009 to 2010. The archived serum samples were examined for the presence of antibodies against *R*. *felis*, *Rickettsia japonica*, and *Rickettsia typhi* using microimmunofluorescence (MIF) assay. Serological evidence of *Rickettsia* exposure was found in 23 (19%, 23/122) patients. Eight patients had antibodies reactive to *R*. *felis*, including four with current infection (a ≥4-fold increase in IgG titer between acute and convalescent sera). The clinical presentations of these four patients included fever, skin rash, lymphadenopathy, as well as more severe conditions such as pancytopenia, hepatomegaly, elevated liver enzymes/bilirubin, and life-threatening acute respiratory distress syndrome. One of the patients died after doxycycline was stopped after being tested negative for scrub typhus, Q fever, and murine typhus.

**Conclusions:**

*Rickettsia felis* is a neglected flea-borne pathogen in Taiwan, and its infection can be life-threatening. Further prospective studies of the prevalence of *R*. *felis* among patients with FUO and compatible clinical manifestations are warranted.

## Author summary

Based on sporadic case reports, *Rickettsia felis* is believed to cause mild infection in humans. However, the true threat of the infection might be underestimated since the laboratory diagnoses of the pathogen were not routinely performed. We conducted a retrospective seroepidemiological survey among patients with clinical symptoms compatible with rickettsioses but negative for scrub typhus, Q fever, and murine typhus at a medical center in Taipei. Immunofluorescence assay was carried out to detect antibodies against *R*. *felis*, *Rickettsia japonica*, and *Rickettsia typhi*. We found that 23 out of 122 (19%, 23/122) such patients were positive for at least one species of rickettsiae, including 4 patients with active *R*. *felis* infection. One of these four patients died after stopping the antibiotic treatment after tested negative for scrub typhus, Q fever, and murine typhus. Our results showed that *R*. *felis* infection is a neglected disease in Taiwan and could be potentially life-threatening.

## Introduction

*Rickettsia felis* is an obligate intracellular bacterium, formerly belonging to the transitional group of *Rickettsia* species [1]. The organism was found sustained in cat flea (*Ctenocephalides felis)*, which is believed to be its primary vector and reservoir, through vertical transmission including transovarial and transstadial transmission [2–5]. Human case of *R*. *felis* infection was described in 1994, and since then, the disease has become an emerging zoonosis reported worldwide [6–14].

The transmission biology of *R*. *felis* is more complicated than previously thought, as more field surveys have detected the bacterial DNA in various arthropods, including fleas, ticks, mites, and mosquitoes [15]. Cat flea is presumed to be the most common vector of *R*. *felis* and transmits the pathogen to vertebrate hosts through infectious bites [16]. Co-migration of humans and domestic animals harboring cat fleas probably contributes to the widespread distribution of *R*. *felis* [17, 18]. Nevertheless, the prevalence of *R*. *felis* within cat fleas fluctuated according to generations and colonies. For example, studies have observed infection rates of 35% to 96% from a single colony over the course of one year and 0 to 100% from F1 progeny for different cat flea colonies [5, 19]. Vertebrate blood source was also proposed to affect the sustainability of *R*. *felis* in cat fleas [3]. Therefore, horizontal amplification or mammalian reservoir hosts are potentially crucial for the maintenance of *R*. *felis* within vector populations. Indeed, horizontal transmission has been demonstrated by cofeeding experiments [16, 20]. A recent report also showed domestic dogs (*Canis familiaris*) were able to sustain prolong periods of rickettsemia [21]. Due to cat flea’s nature of lacking host specificity, several mammals carrying cat fleas, such as opossums, raccoons, and rats have been implicated in the transmission cycle of *R*. *felis*, too [6, 22–26].

*Rickettsia felis* infection causes flea-borne spotted fever (cat flea typhus). The typical clinical presentations included fever, myalgias, arthralgia, nausea, vomiting, hepatitis, photophobia, hearing loss, and other non-specific symptoms [8, 9, 27, 28]. Studies also detected *R*. *felis* in afebrile subjects as well as skin lesions and intact skin [29–32]. *Rickettsia felis* infection has traditionally been characterized as a mild illness compared to other rickettsial diseases. Most patients became afebrile after 3 days of therapy with doxycycline or a fluoroquinolone, and more than 80% of patients had the infection resolved within 7 days [33]. However, neurological symptoms have been reported in Mexico and Sweden, and deaths had been attributed to *R*. *felis* infection based on the evidence of pathogen DNA in the cerebrospinal fluid of two patients presented with meningoencephalitis in Indonesia [8, 14, 34]. Coinfections with other pathogens, such as *Plasmodium*, increased the difficulty of diagnosis [35]. Interestingly, fewer than 15% of patients recalled any exposure to risk animals [2].

In Taiwan, *R*. *felis* infection has been demonstrated to be endemic in cat fleas [36–38]. Sporadic cases of human infection were also reported [13, 39]. However, given that Taiwan Centers for Disease Control (Taiwan CDC) only tested scrub typhus, Q fever, epidemic typhus, and murine typhus for notified suspected rickettsioses, it is likely that cases of *R*. *felis* (and other emerging rickettsioses) had been overlooked or unrecognized [40–45]. Herein, we conducted a retrospective seroepidemiological survey of *R*. *felis* infection among patients with clinically suspected rickettsioses. Microimmunofluorescence (MIF) assay was carried out with archived serum samples to detect antibodies against the pathogen, and medical records of the patients were reviewed.

## Methods

### Ethical statement

The study procedure has been reviewed and approved by the NTUH Research Ethic Committee (REC #201106109RB). The committee waived the need for signed informed consent.

### Study setting and human subjects

**Fig 1:**
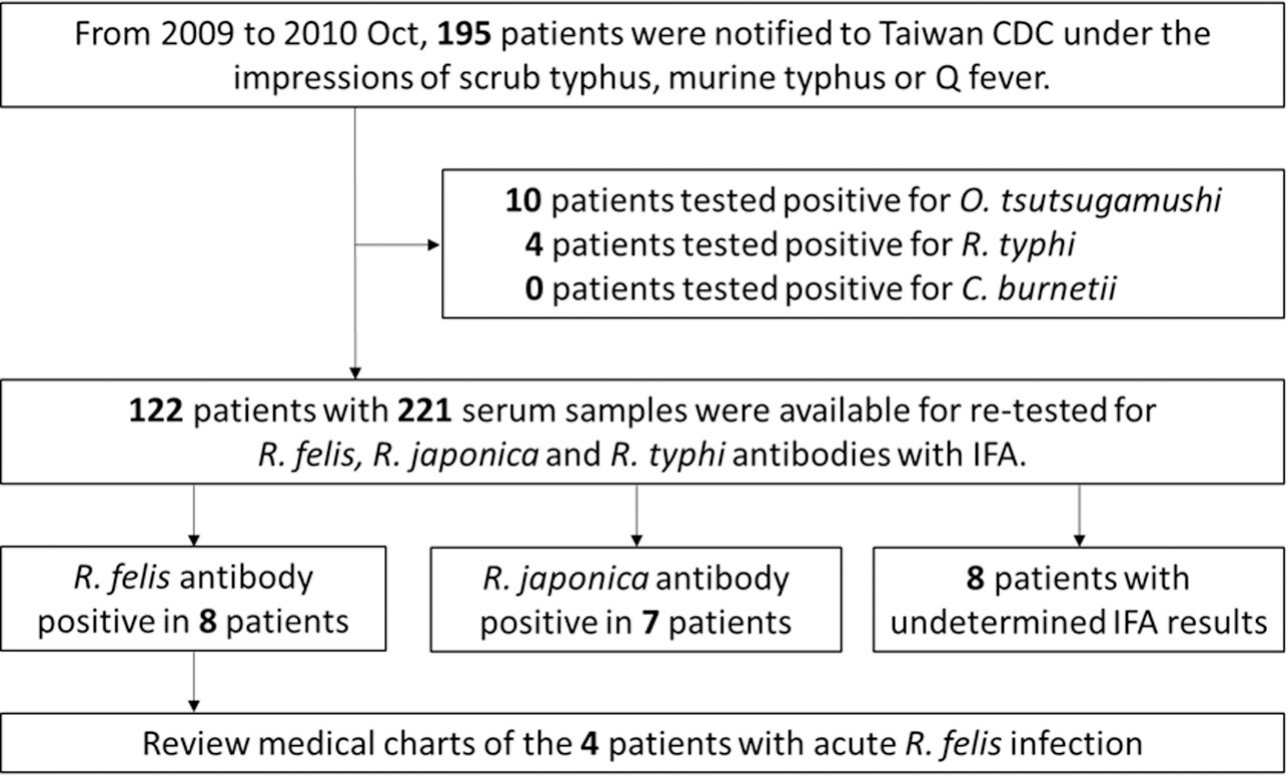
Study design flowchart. The flow diagram showed the experimental design of the study.

National Taiwan University Hospital (NTUH), a 2,800-bed university hospital, provides both primary and tertiary referral care in Taipei, Taiwan. During January 1, 2009 to October 31, 2010, 195 patients presented to NTUH with febrile illness or symptoms resembling those of notifiable rickettsioses, such as persistent high fever, headache, back pain, chills, swollen lymph nodes, rash, eschar, etc. Their blood samples were sent to the Taiwan CDC for laboratory diagnoses. Ten and four patients were diagnosed with scrub typhus and murine typhus, respectively. Of the 181 patients tested negative for scrub typhus, murine typhus, and Q fever, 122 patients gave sufficient amount of blood samples and were included in this retrospective study. Fig 1 illustrated the study design.

### Microimmunofluorescence assay

MIF assay was performed to detect antibodies against *Rickettsia* spp. Besides *R*. *felis*, a combination of antigens was used to help to determine the etiological agent, i.e. *Rickettsia typhi* for typhus group (TG) rickettsiae and *Rickettsia japonica* for spotted fever group (SFG) rickettsiae [46].

Briefly, inactivated whole-cell antigens were dotted on chamber slides and fixed and permeabilized in ice-cold methanol for 10 minutes. The slides were dried and preserved at -80°C until usage. The reactivity of antigens was first verified by banked animal antisera such as cats infested by fleas harboring *R*. *felis* and rodents positive for antibodies against SFG or TG rickettsiae [47]. Then a pool of human serum samples previously tested negative for *R*. *felis*, SFG rickettsiae, and TG rickettsiae antibodies was used as negative control. Sera from 10 patients or individuals with *R*. *felis* and TG rickettsiae infections or SFG rickettsiae antibodies were pooled and served as positive control. The pooled positive and negative control sera were included for each test batch. After reacting with serum sample diluted at 1:64 at 37°C for 30 minutes, slides were washed 3 times in PBS-Tween 20 for 5 min and rinsed with distilled water for a few seconds. Serum antibodies were then labeled with goat anti-human IgG+A+M (H+L)-FITC (Invitrogen, Inc., Waltham, MA, USA) at room temperature in dark for 30 minutes. Unreactive antibodies were washed off by immersing the slides 3 times in PBS-Tween 20 for 5 min. Coverslips were mount onto the slides by mounting media (phosphate buffered saline:glycerol = 3:7) before the slides were examined with a fluorescence microscope (Leica Microsystem, Singapore) by two researchers independently. Images were documented with a SPOT digital camera (RT color, Diagnostic Instruments Inc., Sterling Heights, MI, USA) [48].

For positive samples, further assays were carried out to detect IgM and IgG using specific secondary antibodies. Prior to detection of IgM, sera were treated by rheumatoid factor absorbent (Serion Immundiagnostica GmbH, Wurzburg, Germany). Serum samples were serially diluted to 1:4,096 to determine the titers of antibodies. Patients were considered exposed to the pathogen if (1) IgG titer was ≥1:64 or IgM titer was ≥1:32 for single blood sampling; or (2) seroconversion occurred between paired samples, i.e. a ≥4-fold rise of specific IgG. When cross-reactions occurred, a rickettsial antigen was considered to represent the etiological agent when the antibody titers against this antigen were ≥2-fold higher than the titers of antibodies against other antigens. Otherwise, it would be classified as undetermined infection [49].

### Clinical features

Current/recent infection was defined by exhibiting a ≥4-fold rise in antibody titers between paired serum samples. The medical records of patients who currently/recently infected by *R*. *felis* were retrieved and reviewed. Information about the initial symptoms and signs, clinical course, complications, treatment, and outcomes was analyzed.

## Results

### Exposure to *R*. *felis*, *R*. *typhi*, and *R*. *japonica*

A total of 221 archived serum samples, including 99 paired sera and 23 samples collected either during acute or convalenscent phase, from 122 patients with fever of unknown origin (FUO) were tested by MIF assay. Evidence of rickettsiae exposure was discovered in 23 (19%, 23/122) patients (Table 1). All these patients had antibodies cross-reactive to at least two groups of rickettsial antigens. Eight of the 23 patients were suspected to be infected by *R*. *felis* (patient 1–8), i.e. having antibodies titers against *R*. *felis* ≥2-fold higher than those against *R*. *typhi* or *R*. *japonica*. Four of these patients (patient 1–4) exhibited seroconversion, indicating active *R*. *felis* infection, while the other 4 patients might be infected by *R*. *felis* currently or exposed to the pathogen in the past, which could not be determined from single blood samples. On the other hand, high titer antibodies against *R*. *japonica* were observed in patient 9–15, and the seroconversion in 5 of them (patient 9–13) implied the patients were suffering from infection of SFG rickettsiae. The causative rickettsial agents of the remaining 8 patients (patient 16–23) were undetermined due to the equal antibody titers against two or more groups of rickettsial antigens. In addition, none of these 122 patients showed a higher titer of antibodies against *R*. *typhi* than the other two rickettsial antigens, thus recent *R*. *typhi* infection was not suggested.

**Table 1:** Antibodies against *Rickettsia* spp. in patients with suspected rickettsioses but tested negative for scrub typhus, Q fever, or murine typhus.

Types of rickettsial infection	Patient	1st sampling (days after onset)	2nd sampling (days after onset)	*R*. *felis*				*R*. *typhi*				*R*. *japonica*			
				acute phase		convalescent phase		acute phase		convalescent phase		acute phase		convalescent phase	
				IgG	IgM	IgG	IgM	IgG	IgM	IgG	IgM	IgG	IgM	IgG	IgM
*R*. *felis*	1	9	19	—	—	1,024	—	—	—	64	—	—	—	512	—
	2	7	61	—	—	512	—	—	—	64	—	—	—	128	—
	3	17	28	512	—	2,048	—	—	—	—	—	512	—	512	—
	4	8	18	256	—	1,024	—	—	—	—	—	64	—	128	—
	5	13	NA	NA	NA	1,024	—	NA	NA	64	—	NA	NA	512	—
	6	4	NA	1,024	—	NA	NA	512	—	NA	NA	512	—	NA	NA
	7	32	NA	NA	NA	1,024	—	NA	NA	128	—	NA	NA	512	—
	8	33	NA	NA	NA	512	—	NA	NA	—	—	NA	NA	128	—
Spotted fever group (SFG) rickettsiae	9	2	16	—	—	128	—	—	—	64	—	—	—	1,024	—
	10	6	13	—	—	1,024	—	—	—	256	—	—	—	2,048	—
	11	11	25	—	—	1,024	512	—	—	—	512	—	—	2,048	512
	12	5	25	1,024	—	1,024	—	64	—	512	—	64	—	4,096	—
	13	4	18	—	—	256	—	—	—	—	—	—	—	1,024	—
	14	34	NA	NA	NA	512	—	NA	NA	128	—	NA	NA	1,024	—
	15	3	NA	512	—	NA	NA	256	—	NA	NA	1024	—	NA	NA
Undetermined rickettsial infection	16	8	43	512	512	2,048	256	128	1,024	64	1,024	512	1,024	512	1,024
	17	4	18	—	—	2,048	—	—	—	—	—	—	—	2,048	—
	18	8	17	—	—	512	512	—	—	—	512	—	—	256	512
	19	7	21	—	—	1,024	—	—	—	1,024	—	—	—	1,024	—
	20	98	NA	NA	NA	1,024	—	NA	NA	256	—	NA	NA	1,024	—
	21	4	NA	1,024	1,024	NA	NA	256	256	NA	NA	128	1,024	NA	NA
	22	3	NA	1,024	—	NA	NA	512	—	NA	NA	1,024	—	NA	NA
	23	4	NA	128	128	NA	NA	512	1,024	NA	NA	2,048	512	NA	NA

NA: sample not available

—: negative result

The eight patients potentially exposed to *R*. *felis* were 14 to 77 years of age (median age of 40 years), including 5 males and 3 females (Table 2). Most of them came from northern Taiwan except Patient 1 had a recent travel history to Guangzhou, China. The seasons of onset were mainly winter and spring. None of the patients reported having contact with animals or experiencing flea bites.

**Table 2:** Demographic information of the eight patients with potential *Rickettsia felis* infection.

Blood sample	Patient	Age/Sex	Race	Onset date (yr/mo)	Risk factors				symptoms/signs and lab findings
					Travel history or location	Occupation	Animal contact	Cluster of similar cases	
Paired	1	49/M	Asian	2009/12	Guangzhou, China	businessman	denied	none	fever, muscle ache, skin rashes
	2	47/F	Asian	2010/1	Taipei, Taiwan	hotel housekeeper	denied	none	fever, skin rashes, pancytopenia, abnormal liver function, hepatomegaly
	3	77/M	Asian	2010/3	Hsinchu, Taiwan	retired	denied	none	abnormal liver function
	4	26/F	Asian	2010/4	Taipei, Taiwan	nurse	denied	none	fever, skin rashes, neck lymphadenopathy, abdomen pain
Single blood samples*	5	14/M	Asian	2009/6	Taipei, Taiwan	student	denied	none	fever, skin rashes, consciousness change
	6	29/M	Asian	2009/12	New Taipei City, Taiwan	dining business	denied	none	fever, headache, abnormal liver function
	7	33/F	Asian	2009/11	Taoyuan, Taiwan	business	denied	none	fever
	8	76/M	Asian	2010/3	New Taipei City, Taiwan	retired	denied	none	fever

*The illness episode for which the serum sample was taken may not be caused by active *R*. *felis* infection.

### Clinical features of the *R*. *felis*-infected patients

#### Patient 1

A 49-year-old male returning from Guangzhou, China presented to the physician with fever and myalgias. The patient was admitted after the initial diagnosis of influenza was excluded by the rapid test. The patient developed acute respiratory distress syndrome (ARDS) on the following days and was intubated. Levofloxacin and doxycycline were empirically given for 7 days, and his ARDS gradually improved. Bacterial culture and serology for scrub typhus, Q fever, typhoid fever, and leptospirosis were all negative. Skin rashes developed over axillaries, inguinal area and then progressed to whole lower extremities. Doxycycline was discontinued and methylprednisolone 40 mg per day was started on day 8. Under steroid therapy, however, the patient’s condition deteriorated. Despite intensive supportive care, the patient died from ARDS on day 30. Evidence of *R*. *felis* infection was identified in the study. Antibody titer was increased to 1:1,024 during convalescent phase (Table 1).

#### Patient 2

This 47-year-old woman was admitted due to fever and skin rashes lasting for one week. The patient was a hotel housekeeper and denied other specific travel history or insect bite. Laboratory tests carried out in another hospital found pancytopenia and abnormal liver functions. Abdominal computed tomography showed hepatomegaly without structural lesions. Ceftriaxone and doxycycline were started empirically under tentative diagnosis of scrub typhus or Q fever. Fever subsided on the next days. Complete blood cell count and liver functions gradually returned to normal range. The patient was discharged on day 7 although the serological tests were negative for both scrub typhus and Q fever. MIF assay using paired sera revealed a ≥4-fold increase in IgG titer against *R*. *felis*, suggesting the patient was suffering from *R*. *felis* infection (Table 1).

#### Patient 3

A 77-year-old male, with diabetes mellitus and chronic kidney disease, was admitted for general malaise without fever. *Escherichia coli* bacteremia with septic shock was diagnosed after admission. However, despite intravenous therapy using ceftriaxone actively against *E*. *coli*, the patient’s liver function continued to deteriorate, with elevated aspartate aminotransferase (AST), alanine aminotransferase (ALT), bilirubin (total and direct), alkaline phosphatase (ALP), and gamma-glutamyl transferase (GGT) up to 362 IU/L, 253 IU/L, 7.47 mg/dL, 5.4 mg/dL, 769 IU/L and 386 IU/L, respectively. Abdominal sonography did not reveal structural lesions in liver or gall bladder. Scrub typhus was suspected, but the serological tests gave negative results. A 7-day course of levofloxacin was given. The patient’s liver functions recovered, and the patient was discharged. A ≥4-fold increase in IgG titers against *R*. *felis* was identified between paired sera in the retrospective study (Table 1).

#### Patient 4

A 26-year-old female was admitted due to intermittent fever for one week although one week’s doxycycline had been prescribed as an outpatient. Other accompanied symptoms and signs included skin rashes, abdominal dull pain, and palpable neck lymph nodes. The patient denied any history of travel, occupation, cluster and animal contact. Blood tests revealed elevated AST (205 U/L) and ALT (325 U/L). Viral hepatitis tests and blood culture gave negative results. Doxycycline and flomoxef were given for five days, and her fever subsided on the third day of hospitalization. The patient was then discharged with oral levofloxacin. Scrub typhus was suspected, but the results of both polymerase chain reaction and serological tests were negative. Evidence of *R*. *felis* infection was provided with a ≥4-fold increase in IgG titers between paired sera in the study (Table 1).

## Discussion

We conducted a seroepidemiological survey to retrospectively look for unrecognized *R*. *felis* infection among patients with suspected rickettsioses. We found evidence of *R*. *felis* exposure in 8 patients of whom 4 had been actively infected. The clinical presentations of these 4 patients included not only mild symptoms such as fever, skin rash, or lymphadenopathy in accordance with previous reports but also severe and potential life-threatening conditions including pancytopenia, hepatomegaly, elevated liver enzyme/bilirubin, and acute respiratory distress syndrome. One of the patients (patient 1) died after he was incorrectly treated with intravenous corticosteroid for presumed immunological conditions, after being tested negative for scrub typhus, Q fever, murine typhus, and epidemic typhus. In contrast, patients diagnosed with scrub typhus-associated acute respiratory distress syndrome have been successfully treated with intravenous minocycline [50, 51]. First reported in 2008, sporadic human cases had been documented both in northern and southern Taiwan [13, 39]. In the study, *R*. *felis* infection accounted for up to 21.6% (8/37) of the rickettsioses in 195 patients, suggesting a clinically significant prevalence of this disease in Taiwan. Although most of these patients presented to the hospital in winter and spring a report studying *R*. *felis* in cat fleas parasitizing stray animals in Taipei found a stable infection rate over the course of one year [37]. Our findings highlighted the importance of a correct laboratory diagnosis of rickettsial illnesses. *R*. *felis* infection should be considered as a differential diagnosis for patients present with FUO in the future.

Besides 14 patients diagnosed with scrub typhus and murine typhus by the Taiwan CDC, an additional 23 patients were identified with potential rickettsiae exposure in the study. In another retrospective research study conducted in southern Taiwan involving patients with clinically suspected Q fever, scrub typhus, murine typhus, leptospirosis, and dengue fever, serological evidence of SFG rickettsiae exposure was observed in 18 of 413 patients [39]. However, no indigenous human SFG rickettsioses have been confirmed in Taiwan to date. On the other hand, a variety of SFG rickettsiae has been documented in arthropods and small mammals. Novel species including *Rickettsia* sp. TwKM01, *Rickettsia* sp. IG-1, *Rickettsia* sp. RR01were isolated from *Rhipicephalus haemaphysalodies*, *Ixodes granulatus*, and *Rhipicephalus sanguineus* ticks, and *Rickettsia* sp. TwKM02 was isolated from *Leptotrombidium deliense* mites [38, 44, 45]. *Rickettsia conorii*, *R*. *japonica*, *Rickettsia rickettsii*, *Rickettsia australis*, *Rickettsia helvetica*, *Rickettsia monacensis* have been detected in fleas and ticks collected from small mammals and birds [43, 47, 52, 53]. Infections of *R*. *conorii*, *R*. *japonica*, *R*. *rickettsii*, *Rickettsia* sp. TwKM01, *Rickettsia* sp. IG-1, *Rickettsia* sp. TwKM02, or *Rickettsia raoultii* were also demonstrated in rodents by molecular analysis or serology [47, 53, 54]. Based on the concept of “One Health”, the diverse and prevalent SFG rickettsiae in the field would increase the potential risk for human SFG rickettsioses. Moreover, MIF assay yielded positive results against at least 2 species of *Rickettsia* with equal titers in 8 patients, thus the etiological agents were undetermined in the study. The patients could be infected either by more than one species of *Rickettsia* simultaneously/sequentially or by unknown agents which cross-reacted with two groups of antigens. Surveillance of *Rickettsia* spp. in the field and febrile patients should be taken continuously to detect possible threats in order to control the infections.

In the study, the serological results did not suggest recent *R*. *typhi* infection in any of the 122 patients presenting to NTUH in Taipei. This might be because murine typhus occurs sporadically in Taiwan, and most cases are located in the southwest and central-west districts, including Kaohsiung-Pingtung region, Tainan City, Changhua County, and Taichung City [55]. Although extensive cross-reaction of antibodies happened between *R*. *typhi* and *Rickettsia prowazekii* [56], the latter has not been reported in Taiwan since World War II.

There were certain limitations in our study. Although molecular methods offer better sensitivity and specificity for diagnosis of acute rickettsial infections, DNA samples from acute phase PBMC or whole blood before administration of antibiotics and autopsy specimens were unavailable in the retrospective study. We were unable to isolate the pathogens or provide direct molecular evidence of infections. Nevertheless, periods of rickettsemia may quickly subside. The serological reference method for rickettsioses is immunofluorescence analysis. Best efforts were made to retrieve paired samples (acute and convalescent) drawn approximately 2 weeks apart from the study subjects, but in some cases, samples were not collected in the recommended time frame, or only single serum samples were available (Table 1). Since some patients were referred to NTUH, the first sampling times have past 7 days after onset, however, their symptoms were not resolved while administration. Significant changes in antibody titers against *R*. *felis* and *R*. *japonica* were observed in 4 patients (patient 1–4) and 5 patients (patient 9–13), respectively, suggesting recent infections. On the other hand, for patients with single samples, a single titer result does not differentiate between past and current exposure. They might presented to the hospital due to other acute infections. MIF assay allowed the detection of antibodies against different rickettsial antigens within the same drop of diluted serum at the same time. Difference in antibody titers was used to distinguish cross-reactions between groups of rickettssial antigens, but cross-reactions between closely related species of *Rickettsia* could not be ruled out without the use of further serological assays, such as cross-adsorption technique and western blot. Therefore, from a critical point of view, our findings of anitbodies against *R*. *felis* in patients may be in fact caused by infection of organisms closely related to *R*. *felis*.

In conclusion, *R*. *felis* is a neglected zoonotic pathogen in Taiwan, and without properly treatment, the infection can be lethal. Considering the accumulating reports of detected SFG rickettsiae in animals and arthropods in the field, human rickettsioses are likely to happen more frequently than previously thought. This work highlighted the importance of a correct and prompt laboratory diagnosis. Inclusion of *R*. *felis* infection in routine differential diagnosis would help to guide appropriate treatment. Further prospective studies of the emerging rickettsiosis among FUO patients presented with compatible clinical manifestations are warranted.
