# Supplementary material for: Neglected human Rickettsia felis infection in Taiwan: A retrospective seroepidemiological survey of patients with suspected rickettsioses
Source: PLoS Negl Trop Dis. 2021 Apr 19;15(4):e0009355. doi: 10.1371/journal.pntd.0009355 (PMC8084327; doi:10.1371/journal.pntd.0009355)
Supplement: Attachment — Submitted filename: Peer Review Article - Rickettsia felis.docx [file pntd.0009355.s001.docx]

Peer Review Article

This exciting paper by Wang-Hsiu Yang and co-workers examines the seroprevalence of *Rickettsia felis* infection in Taiwan. The authors examine 122 patients with fever of unknown origin and describe the clinical outcomes of 4 patients with current *R. felis* infection. The paper submitted is an important and valuable study that sets *R. felis* as a pathogen present in Taiwan with potentially fatal outcomes.

Line 95 states that no serious complications or death have been reported with *R. felis*. While *R. felis* infection has traditionally been characterized as a mild infection compared to other rickettsial diseases, *R. felis* has been found in the CSF of patients and has been attributed to two fatal cases of meningoencephalitis in humans (PMID: 32069292). Furthermore, this pathogen has been reported to have generalized CNS involvement in severe illness (PMID: 11009147 and PMID: 24188709).

The paper has a few minor editing issues:

Line 72 - I believe should be “field” survey’s

Line 84 – “A” recent report…

Line 96 – Interestingly, “fewer” than, instead of lesser than

Line 98 – should probably be demonstrated to be endemic in cat fleas.

Line 253 – “an” additional 23 patients...

Line 254 – research “study” conducted in…

The authors have provided no details on positive or negative controls in the materials/methods for MIF assays. While I understand molecular analysis of blood samples is not possible in a retrospective study like this, it would undoubtedly add to the paper’s overall impact, especially in the fatal case described for patient 1. Autopsy specimens are likely available and would provide DNA for sequencing.

Serological cross-reactivity is a well-documented issue in rickettsial diagnosis. While the author’s state (starting on line 295) that patients may be infected with similar rickettsia species, it is speculative to attribute *R. felis* fatality based on serology alone. A description of the histopathology from case 1 would add to this paper, though it is still not enough to attribute death from rickettsiae based on the data provided.
