# Supplementary material for: Neglected human Rickettsia felis infection in Taiwan: A retrospective seroepidemiological survey of patients with suspected rickettsioses
Source: PLoS Negl Trop Dis. 2021 Apr 19;15(4):e0009355. doi: 10.1371/journal.pntd.0009355 (PMC8084327; doi:10.1371/journal.pntd.0009355)
Supplement: Attachment — Submitted filename: Response to reviewers_20200215.docx [file pntd.0009355.s002.docx]

**Reviewer #1**

- Methods. The objectives of this study are clearly defined, and the design is appropriate for the stated objectives. The patient population from which this cohort was derived is described but how the patients were identified is lacking detail. The sample size and analysis were appropriate for this descriptive study. Ethical requirements were met.

**Authors’ response:** During Jan 1^st^, 2009 to Oct 31^st^, 2010, 195 patients were presented to NTUH with symptoms resembling those of notifiable rickettsioses in Taiwan. Their blood samples were therefore sent to the Taiwan CDC for laboratory diagnoses following the regulations. The test results showed 14 patients were infected by *Orientia tsutsugamushi* or *Rickettsia typhi*. The residual serum samples from 59 patients were not available, or the volume was not sufficient for the investigation. Therefore, only samples from the remaining 122 patients were examined in the study (**L121-128**).

- Results. The conclusion is supported by the data. Limitations are presented but I believe that further limitations of serologic testing should be discussed. Specifically, the reliance on a single point titer cannot distinguish acute and historic infection.

**Authors’ response:** Indeed, only 4 patients with seroconversion indicated active infection, but the other 4 patient might be exposed to *R. felis* in the past, which cannot be determined based on single blood samples. The point has been addressed in the manuscript (**L187-188, Table 1**).

- Editorial and Data Presentation Modifications. Please include a presentation of the epidemiological data for the 9 R. felis case-patients (i.e., age, gender, race, moth and year of onset, location of case-patient).

**Authors’ response:** The epidemiological data for the 8 patients with potential *R. felis* exposure were described, and a table was added according to the reviewer’s suggestion (**L200-204, Table 2**).

- Summary and General Comments. The manuscript titled “Neglected human Rickettsia felis infection in Taiwan: a retrospective seroepidemiological survey of patients with suspected rickettsioses” is a well written and important preliminary analysis of the public health impact of R. felis in Taiwan. I believe the analysis presented is appropriate for the objectives of the study. I think an epidemiologic presentation of the cases is missing from this analysis. Specifically, describing the demographics of the R. felis case-patients, the temporality of the case, and the geographic distribution of the cases. This information may be important for public health officials to aid in directing testing in the future. I have some additional specific comments listed below:

**Authors’ response:** The epidemiological data for the 8 *R. felis* case-patients were described in Table 2. (**L200-204, Table 2**). Since our study site, NTUH, is the biggest hospital located in Taipei, most of the patients came from northern Taiwan. Only one patient (Patient 1) returned from mainland China. Moreover, although most of these patients had symptom onset in winter and spring, our previous study on infection rate of *R. felis* in cat fleas parasitizing stray animals did not reveal statistically significant change over the course of one year (**L271-273**).

- Line 118. What symptoms were used as criteria for selection? How were the patients identified?

**Authors’ response:** 195 patients clinically suspected with notifiable rickettsioses were reported to the Taiwan CDC from NTUH, but 181 patients gave negative results. Among the 181 patients, only 122 patients had sufficient residual serum samples for the tests in the study. The symptoms of rickettsial infections included persistent high fever, headache, back pain, chills, swollen lymph nodes, rash, eschar, etc. (**L121-128**)

- Table 1. I believe the authors should consider adding discussion of timing of the acute and convalescent sample. Not all samples were collected in the recommended time frame for acute and convalescent testing.

**Authors’ response:** A discussion about timing of the samples has been added in the manuscript as suggested (**L315-322**). Despite our efforts to collect paired samples, some patients presented to NTUH after 7 days post symptom onset, especially patients who were referred from other clinics.

- Line 284. An important limitation to include is the use of one time point serologic testing. Those with only one time point cannot be determined as acutely infected, especially patient 6 who was sampled only 4 days after symptom onset. You cannot rule out that these were historic cases presenting with other etiology acute infection.

**Authors’ response:** A discussion about the interpretation of test results from paired sera and single serum samples was added (**L322-325**).

- Table 1. why is patient 18 considered unspecified? There is a 4-fold increase in R. felis IgG titers and the convalescent titer is 2-fold higher than others?

**Authors’ response:** 4-fold increases were observed in all 3 rickettsiae IgG with equal titers for patient 18 (**Table 1**). As for patient 16, although a 4-fold increase in *R. felis* IgG titers and the convalescent titer is 2-fold higher than others, somehow, the IgM titers for *R. typhi* and *R. japonica* remained higher than *R. felis*. Therefore, from a conservative point of view, we categorized the infection was caused by an undetermined etiological agent or a coinfection.

**Reviewer #2**

- This exciting paper by Wang-Hsiu Yang and co-workers examines the seroprevalence of Rickettsia felis infection in Taiwan. The authors examine 122 patients with fever of unknown origin and describe the clinical outcomes of 4 patients with current R. felis infection. The paper submitted is an important and valuable study that sets R. felis as a pathogen present in Taiwan with potentially fatal outcomes.
- Line 95 states that no serious complications or death have been reported with R. felis. While R. felis infection has traditionally been characterized as a mild infection compared to other rickettsial diseases, R. felis has been found in the CSF of patients and has been attributed to two fatal cases of meningoencephalitis in humans (PMID: 32069292). Furthermore, this pathogen has been reported to have generalized CNS involvement in severe illness (PMID: 11009147 and PMID: 24188709).

**Authors’ response:** Thanks for the update. The references have been added in the manuscript (**L96-100, L450-458**).

- The paper has a few minor editing issues:

Line 72 - I believe should be “field” survey’s

**Authors’ response:** The typo has been corrected (**L72**).

Line 84 – “A” recent report…

**Authors’ response:** A numeral word has been added (**L84**).

Line 96 – Interestingly, “fewer” than, instead of lesser than

**Authors’ response:** The “lesser” has been replaced by “fewer” in the sentence (**L101**).

Line 98 – should probably be demonstrated to be endemic in cat fleas.

**Authors’ response:** The sentence has been modified accordingly (**L102**).

Line 253 – “an” additional 23 patients...

**Authors’ response:** The sentence has been corrected (**L278**).

Line 254 – research “study” conducted in…

**Authors’ response:** The sentence has been modified (**L279**).

- The authors have provided no details on positive or negative controls in the materials/methods for MIF assays. While I understand molecular analysis of blood samples is not possible in a retrospective study like this, it would undoubtedly add to the paper’s overall impact, especially in the fatal case described for patient 1. Autopsy specimens are likely available and would provide DNA for sequencing.

**Authors’ response:** Details on positive and negative controls have been added in the materials/methods for MIF assays (**L141-148**). Pools of sera previously tested negative and positive for antibodies against *R. felis*, SFG rickettsiae, and TG rickettsiae were used as negative control and positive control, respectively. We agree histopathology would have provided stronger evidence and details about the pathogenesis. Unfortunately, this patient didn’t receive autopsy (**L312**).

- Serological cross-reactivity is a well-documented issue in rickettsial diagnosis. While the author’s state (starting on line 295) that patients may be infected with similar rickettsia species, it is speculative to attribute R. felis fatality based on serology alone. A description of the histopathology from case 1 would add to this paper, though it is still not enough to attribute death from rickettsiae based on the data provided.

**Authors’ response:** As mentioned in the manuscript, the possibility of cross-reaction still couldn’t be ruled out between closely related species of *R. felis* by MIF, e.g. *R. felis*-like organisms (RFLOs). Unfortunately, for this retrospective study, we were unable to retrieve PBMC or autopsy specimens. The infection of *R. felis* can only be determined by the clinical symptoms, responses to antibiotic therapy, and serology. We couldn’t be certain that the fatality was directly associated with *R. felis* infection. We therefore described the event according to the observations. One of the strongest evidence of *Rickettsia* infection was that the patient’s condition deteriorated after stopping antibiotics and eventually died from ARDS (**L216-219**).
